# Limited *ERG11* Mutations Identified in Isolates of Candida auris Directly Contribute to Reduced Azole Susceptibility

**DOI:** 10.1128/AAC.01427-18

**Published:** 2018-09-24

**Authors:** Kelley R. Healey, Milena Kordalewska, Cristina Jiménez Ortigosa, Ashutosh Singh, Indira Berrío, Anuradha Chowdhary, David S. Perlin

**Affiliations:** a Public Health Research Institute, New Jersey Medical School, Rutgers Biomedical and Health Sciences, Newark, New Jersey, USA; b Department of Medical Mycology, Vallabhbhai Patel Chest Institute, University of Delhi, Delhi, India; c Medical and Experimental Mycology Group, Corporación para Investigaciones Biológicas (CIB), Medellín, Colombia; d Hospital General de Medellin Luz Castro de Gutiérrez ESE, Medellín, Colombia

**Keywords:** Candida auris, *ERG11*, antifungal resistance, azoles, reduced azole susceptibility

## Abstract

Multiple Erg11 amino acid substitutions were identified in clinical isolates of Candida auris originating from India and Colombia. Elevated azole MICs were detected in Saccharomyces cerevisiae upon heterologous expression of C. auris *ERG11* alleles that encoded for Y132F or K143R substitutions; however, expression of alleles encoding I466M, Y501H, or other clade-defined amino acid differences yielded susceptible MICs. Similar to other Candida species, specific C. auris *ERG11* mutations resulted directly in reduced azole susceptibility.

## Abstract (precis)

Multiple Erg11 amino acid substitutions were identified in clinical isolates of Candida auris originating from India and Colombia. Elevated azole MICs were detected in Saccharomyces cerevisiae upon heterologous expression of C. auris *ERG11* alleles that encoded for Y132F or K143R substitutions; however, expression of alleles encoding I466M, Y501H, or other clade-defined amino acid differences yielded susceptible MICs.

## TEXT

The emerging pathogen Candida auris has spread across the globe, caused hospital outbreaks, and been reported as refractory to common antifungal agents, including triazoles, such as fluconazole and voriconazole. Currently, C. auris is divided into four major clades: South Asian, East Asian, South African, and South American (1). C. auris-related infections in other parts of the world, such as the United States or United Kingdom, have been caused by strains that are genetically related to these clades (2, 3). Transmission of highly clonal C. auris isolates within health care facilities has triggered institutional outbreaks, further emphasizing the importance of understanding resistance mechanisms in this yeast. Here, we determined azole susceptibilities and *ERG11* genotypes from Indian (South Asian clade) and Colombian (South American clade) isolates and subsequently evaluated the significance of specific *ERG11* mutations and their potential ability to confer azole resistance.

Multiple mechanisms of azole resistance have been described in Candida albicans, including mutations in the ergosterol synthesis pathway (primarily in the azole target *ERG11*), upregulation of *ERG11*, and upregulation of drug efflux pumps (e.g., *CDR1*, *CDR2*, *MDR1*) due to a gain in function mutations in transcription factors (e.g., *TAC1*, *MRR1*) that induce their expression (4). In 2017, Lockhart and colleagues (1) identified Erg11 amino acid substitutions (e.g., F126T, Y132F, K143R) in isolates of C. auris from South Africa (South African clade), Venezuela (South American clade), and India and Pakistan (South Asian clade). Of note, the F126 substitution identified in South African isolates has since been described as F126L (3, 5). These substitutions were associated with elevated azole MICs (1). Additionally, Chowdhary and colleagues (6) recently identified Erg11 amino acid substitutions Y132F and K143R in 100% (34/34) of C. auris isolates from India that demonstrated elevated fluconazole MICs (32 to ≥64 μg/ml). Notably, a wild-type *ERG11* genotype was reported in 4 of 5 isolates exhibiting low fluconazole MICs (1 to 2 μg/ml) (6). The *ERG11* gene is highly conserved among Candida species, and specific Erg11 substitutions in C. albicans, including F126L, Y132F, and K143R, are directly associated with resistance (see reference 7 for a comprehensive review of characterized mutations) and have been shown to exhibit reduced susceptibilities to azoles upon heterologous expression in Saccharomyces cerevisiae (8–10). These expression studies were later verified through direct expression in C. albicans (11). The equivalent Y132F and/or K143R substitutions were also associated with azole resistance in Candida tropicalis (12–14), Candida parapsilosis (15, 16), and Candida orthopsilosis (17). Residues Tyr132 and Lys143 lie within the substrate binding pocket of the Erg11 protein (lanosterol 14α-demethylase) and have been shown in S. cerevisiae to directly interact with and stabilize the binding of fluconazole (18). As anticipated, mutation of these residues adversely affects the binding of fluconazole, resulting in reduced susceptibility (18).

We sequenced the *ERG11* coding regions from 40 isolates originating from Vallabhbhai Patel Chest Institute in Delhi, India, and 56 isolates originating from Clinica General del Norte in Barranquilla, Colombia, and a tertiary care center in Santa Marta, Colombia (see Table S1 in the supplemental material for primers). Antifungal susceptibility testing was performed according to CLSI methodology (19). As previously reported, nearly all of the isolates from India demonstrated elevated fluconazole and voriconazole MICs and contained a Y132F or K143R Erg11 substitution (Table 1) (6). One isolate from the same geographic area did not contain an amino acid difference (Erg11-wild type) and exhibited susceptible MICs (Table 1). All isolates originating from Colombia contained the same three Erg11 substitutions (K177R, N335S, E343D), in contrast to the Indian isolates, and likely represent polymorphic clade differences (see below). Overall, the Colombian isolates were more susceptible to azoles than the Indian isolates, although we did identify 3 Colombian isolates that contained Y132F or K143R and exhibited higher MICs, as expected (Table 1). Out of the 35 otherwise wild-type Colombian isolates, only 4 demonstrated elevated MICs (≥32 and ≥0.5 μg/ml for fluconazole and voriconazole, respectively). The second-most prevalent *ERG11* allele identified within the Colombian isolates encoded an I466M amino acid substitution (Table 1). Interestingly, these isolates also demonstrated a wide range of fluconazole and voriconazole MICs, including 4 strains with a fluconazole MIC of 32 μg/ml. A single isolate exhibited a Y501H substitution and decreased azole susceptibilities (Table 1). While the Erg11 amino acid numbering is consistent between C. auris and C. albicans for Y132 and K143, C. auris I466 and Y501 are equivalent to C. albicans I471 and Y505, respectively.

**TABLE 1:** Identified C. auris Erg11 variants and associated azole MICs, origin, and frequency

Erg11 substitution^*a*^	MIC range (mode)^*b*^ in µg/ml		Origin (no. of isolates)
	Fluconazole	Voriconazole	
Wild type	4	0.03	India (1)
Y132F	64 to >128 (>128)	4 to >16 (4)	India (22)
K143R	64 to >128 (>128)	0.5 to 1 (0.5)	India (17)
Wild type	2 to 64 (2)	0.03 to 1 (0.06)	Colombia (35)
Y132F	>128	2 to 4	Colombia (2)
K143R	32	0.12	Colombia (1)
I466 M	4 to 32 (16)	0.06 to 0.5 (0.12)	Colombia (17)
Y501H	64	1	Colombia (1)

a All Colombian isolates exhibited K177R, N335S, and E343D polymorphisms, in contrast to Indian isolates.

b Mode shown for genotypes found in ≥10 isolates.

To better assess the significance of specific *ERG11* mutations in azole susceptibility, we cloned each identified *ERG11* allele, including the promoter region, onto a low-copy CEN/ARS-containing plasmid (pRS416) with direct transformation into S. cerevisiae through gap-repair cloning as previously described (20) (see Table S1). Because C. auris is a haploid organism, we utilized the S. cerevisiae BY4741 haploid strain (3). Transformants were selected on synthetic-defined medium lacking uracil (SD-ura) and screened by PCR for correct insertion. For each transformation, multiple PCR-positive colonies were passaged on SD-ura medium and plasmid inserts sequenced to confirm *ERG11* genotypes. MICs were performed in both nutrient-rich (yeast extract, peptone, dextrose [YPD]) and nutrient-limited (SD-ura) media, and results were consistent (≤2-fold changes) between the two (Table 2). S. cerevisiae that expressed C. auris Erg11-Y132F or Erg11-K143R exhibited elevated MICs to fluconazole (64 to 128 μg/ml) and voriconazole (0.5 to 2 μg/ml), while expression of the wild-type allele or empty vector demonstrated susceptible MICs (≤16 μg/ml to fluconazole, ≤0.12 μg/ml to voriconazole) (Table 2). Additionally, susceptible MICs were detected upon expression of the Colombian *ERG11* wild-type allele (K177R, N335S, E343D) and alleles encoding the I466M or Y501H substitutions (Table 2).

**TABLE 2:** Azole susceptibilities of S. cerevisiae expressing C. auris *ERG11* alleles

Plasmid^*a*^	MIC (μg/ml)			
	Fluconazole		Voriconazole	
	YPD	SD-ura	YPD	SD-ura
Empty vector	8	8	0.12	0.12
CauErg11-wild type (India)	16	8	0.12	0.12
CauErg11-wild type (Colombia)	8	8	0.12	0.12
CauErg11-Y132F	128	128	2	1
CauErg11-K143R	64	32	0.5	0.25
CauErg11-I466M	8	8	0.12	0.12
CauErg11-Y501H	8	8	0.12	0.06

a Colombian sequences also contained K177R, N335S, and E343D polymorphisms.

In fact, K177R, N335S, and E343D amino acid substitutions were found in all Colombian isolates and did not contribute to any decrease in azole susceptibility. These changes likely represent genetically evolved clade differences. Reduced susceptibilities to azoles were detected in 8 Colombian isolates that exhibited either wild-type or I466M Erg11 sequences and in the sole Y501H isolate. Susceptible MICs were detected in our S. cerevisiae cloning assay for these variants (Table 2), indicating that the I466M and Y501H Erg11 substitutions alone do not impart resistance. Interestingly, substitution of the amino acid equivalent to I466 in C. albicans (I471T) has been reported in 2 resistant isolates of C. albicans (21, 22), although elevated MICs were only observed following overexpression (high-copy-number plasmid) of this *ERG11* allele in S. cerevisiae, whereas expression on a low-copy vector resulted in susceptible MICs, reflecting the influence of a gene dosage effect (21). Note that in one case, this mutation in C. albicans was found in combination with Y132H (21). It is possible that I466M, and potentially Y501H, contribute to a modest decrease in azole susceptibility in C. auris, but this would be dependent on an increase in *ERG11* expression. Although we did not measure *ERG11* gene expression in our S. cerevisiae strains, we concluded that our constructs did not have significant effects on gene dosage, as expression of the wild-type and the nonresistance-conferring *ERG11* alleles exhibited MICs (1- to 2-fold) similar to those of the empty-vector control strain (Table 2).

In conclusion, the Indian isolates demonstrated greater rates of triazole resistance than the Colombian isolates analyzed here. Only the Y132F and K143R Erg11 amino acid substitutions identified in both sets of isolates were independently confirmed to directly mediate reduced azole susceptibility. The Y132F substitution led to the most pronounced reduction in azole susceptibility. Mechanisms other than *ERG11* mutation (e.g., *ERG11* overexpression or efflux pumps), particularly in the South American clade, may also contribute to reduced azole susceptibility in C. auris, although this remains to be determined. Mutations leading to the Y132F and K143R substitutions may be valuable as initial molecular markers for C. auris azole resistance in South Asian and South American clade isolates.

## Supplementary Material

Supplemental file 1
