# Supplementary material for: Limited ERG11 Mutations Identified in Isolates of Candida auris Directly Contribute to Reduced Azole Susceptibility
Source: Antimicrob Agents Chemother. 2018 Sep 24;62(10):e01427-18. doi: 10.1128/AAC.01427-18 (PMC6153782; doi:10.1128/AAC.01427-18)
Supplement: Supplemental file 1 [file zac010187533s1.pdf]

Supplementary table 1. Primers used in this study.

Primer*	Application	Sequence (5’-3’)†
CauERG11u376F	PCR	GCTCGGTTATCTGCTGACTC
CauERG11d176R	PCR	GGAACCTTCAAGCTGAACTTG
CauERG11c202R	sequencing	GCTGCATTCCGTAAACAACAG
CauERG11c582R	sequencing	CTCAGGCTGAGTCTTCATC
CauERG11c1215R	sequencing	GGAGACCATCACGTAGTGAC
CauERG11d176R	sequencing	GGAACCTTCAAGCTGAACTTG
pRS-CauERG11F	gap-repair cloning	GGCCCCCCCTCGAGGTCGACGGTATCGATAAGCTTGATATCGAATTCCTGCAGGCTCGGTTATCTGCTGACTC
pRS-CauERG11R	gap-repair cloning	CAAAAGCTGGAGCTCCACCGCGGTGGCGGCCGCTCTAGAACTAGTGGATCCGGAACCTTCAAGCTGAACTTG
CauERG11c202R	PCR screen	GCTGCATTCCGTAAACAACAG
T7	PCR screen	GCGTAATACGACTCACTATAGG
T3	PCR/sequencing	AAGCGCGCAATTAACCCTCAC

*****Numbers in primer names correspond to nucleotide location upstream (u) or within the coding region (c) relative to the start codon, or downstream (d) relative to the stop codon.
†Underlined regions of gap-repair cloning primers correspond to *C. auris ERG11* upstream or downstream sequences, and non-underlined regions correspond to sequences on pRS416 surrounding SmaI restriction site.
